# Plasma genetic and genomic abnormalities predict treatment response and clinical outcome in advanced prostate cancer

**DOI:** 10.18632/oncotarget.3845

**Published:** 2015-04-15

**Authors:** Shu Xia, Manish Kohli, Meijun Du, Rachel L. Dittmar, Adam Lee, Debashis Nandy, Tiezheng Yuan, Yongchen Guo, Yuan Wang, Michael R. Tschannen, Elizabeth Worthey, Howard Jacob, William See, Deepak Kilari, Xuexia Wang, Raymond L. Hovey, Chiang-Ching Huang, Liang Wang

**Affiliations:** ^1^ Department of Oncology, Tongji Hospital of Tongji Medical College, Huazhong University of Science and Technology, Wuhan, China; ^2^ Department of Pathology and MCW Cancer Center, Medical College of Wisconsin, Milwaukee, WI, USA; ^3^ Department of Medical Oncology, Mayo Clinic, Rochester, MN, USA; ^4^ Molecular Pharmacology and Experimental Therapeutics, Mayo Clinic, Rochester, MN, USA; ^5^ Human Molecular Genetics Center, Medical College of Wisconsin, Milwaukee, WI, USA; ^6^ Department of Urology, Medical College of Wisconsin, Milwaukee, WI, USA; ^7^ Joseph J. Zilber School of Public Health, University of Wisconsin, Milwaukee, WI, USA; ^8^ Great Lakes Genomics Center, School of Freshwater Sciences, University of Wisconsin, Milwaukee, WI, USA

**Keywords:** prostate cancer, liquid biopsy, plasma, cell free DNA, next generation sequencing

## Abstract

Liquid biopsies, examinations of tumor components in body fluids, have shown promise for predicting clinical outcomes. To evaluate tumor-associated genomic and genetic variations in plasma cell-free DNA (cfDNA) and their associations with treatment response and overall survival, we applied whole genome and targeted sequencing to examine the plasma cfDNAs derived from 20 patients with advanced prostate cancer. Sequencing-based genomic abnormality analysis revealed locus-specific gains or losses that were common in prostate cancer, such as 8q gains, *AR* amplifications, *PTEN* losses and *TMPRSS2-ERG* fusions. To estimate tumor burden in cfDNA, we developed a Plasma Genomic Abnormality (PGA) score by summing the most significant copy number variations. Cox regression analysis showed that PGA scores were significantly associated with overall survival (*p* < 0.04). After androgen deprivation therapy or chemotherapy, targeted sequencing showed significant mutational profile changes in genes involved in androgen biosynthesis, *AR* activation, DNA repair, and chemotherapy resistance. These changes may reflect the dynamic evolution of heterozygous tumor populations in response to these treatments. These results strongly support the feasibility of using non-invasive liquid biopsies as potential tools to study biological mechanisms underlying therapy-specific resistance and to predict disease progression in advanced prostate cancer.

## INTRODUCTION

Androgen deprivation therapy (ADT) has been used to treat advanced prostate cancer since 1941 [1]. In 2011, more than one-third of the estimated 2.71 million prostate cancer patients in the United States received ADT (http://seer.cancer.gov/). Response to ADT in the hormone-sensitive prostate cancer (HSPC) lasts from a few months to several years (median 18-30 months). To date, there are no known predictive factors for duration of ADT response. After the emergence of castration-resistant prostate cancer (CRPC), several new systemic anti-cancer therapies with overall survival benefit are currently considered [2]. A biochemical response to these treatments is often estimated by PSA levels. However, this estimate may be unreliable due to disease heterogeneity. Development of more sensitive and specific assays to monitor the treatment response is clearly needed.

Traditional biopsies use solid tumor tissues to assess genomic architecture. However, multiple or serial traditional biopsies can be impractical because they are potentially hazardous to patients and technically challenging. Recently, the assessment of tumor-released DNA in body fluids such as cell-free DNAs (cfDNAs) in plasma has shown promise in being able to capture the net effect of the host-tumor genetic fraction in cancer patients [3-5]. Critically, whole genome sequencing has revealed significant copy number variations (CNVs) both in somatic tumor tissues as well as in the cfDNA fractions of cancer patients [6-10]. As a result of the accessibility of sampling as well as the ability to capture the genetic heterogeneity of cancer in peripheral fluids, there has been a growing interest in developing tumor-derived cfDNA as a biomarker for detecting the presence of malignancies, monitoring treatment response, judging prognosis, or evaluating recurrence [3-19]. The examination of tumor components including circulating tumor cells and nucleic acids such as cfDNA in body fluids is often referred to as a liquid biopsy [3-5].

To determine tumor-related genomic abnormalities in plasma cfDNAs and their association with treatment response and clinical outcome, we performed whole genome sequencing-based CNV and targeted sequencing-based mutational analysis in cfDNAs derived from patients with advanced prostate cancer. To precisely reflect tumor burden and estimate treatment response, we developed two scoring algorithms based on a composite score from the cfDNA genomic abnormality profiles. Our results demonstrate that non-invasive liquid biopsy technology is feasible and has potential to serve as a powerful tool for personalized management of advanced prostate cancer.

## RESULTS

### Overall cfDNA genomic abnormality in advanced prostate cancer patients

We examined three samples for each patient including pre-treatment cfDNA, post-treatment cfDNA and matched lymphocyte-derived germline DNA (gDNA). Whole genome sequencing generated approximately14.48 million (ranged from 9.19 to 21.72) mappable reads per sample and ~4,560 mappable reads per genomic bin window (1Mb). CNV analysis using log2 ratios between cfDNA and matched gDNA showed somatic genomic abnormalities in all 20 patients tested. Overall, we observed more genomic abnormalities in the CRPC cohort undergoing chemotherapy than in the HSPC cohort receiving ADT alone (Supplementary Figure S1).

**Figure 1:**
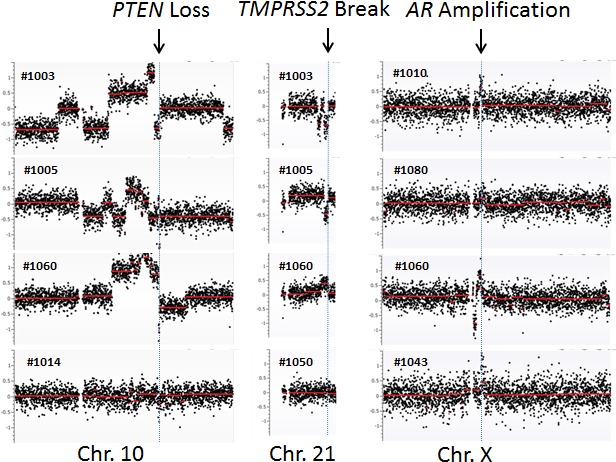
cfDNA genomic abnormalities detected at specific chromosomal loci *PTEN* loss at chromosome 10, *TMPRSS2* at chromosome 21, and *AR* amplification at chromosome X are shown. Arrows indicate the locations of these chromosomal aberrations.

To further define the CNVs, we performed a detailed analysis at chromosomal regions showing frequent aberrations in prostate cancer. Among these, the genomic region at the androgen receptor (*AR*) was most frequently reported to be amplified [20, 21]. To examine the amplification status, we zoomed into the genomic region containing *AR* and observed *AR* locus amplification in 1 of 10 HSPC (#1080) and 3 of 10 CRPC cases (#1010, #1043 and #1060) (Figure 1). Another common genomic aberration in prostate cancer was various fusion genes at the *TMPRSS2* locus [22, 23]. We observed two CRPC patients (#1003 and #1005) with genomic loss and two patients with genomic gain - one CRPC patient (#1060) and one HSPC patient (#1050). Both genomic losses resulted in the *TMPRSS2-ERG* fusion gene (Figure 1). Interestingly, the genomic loss at the *TMPRSS2* locus was present in two CRPC patients with a pathological diagnosis of small cell carcinoma (neuro-endocrine origin). These two patients did not show *AR* amplification. The third most common genomic abnormality was *PTEN* deletion [24, 25], which was detected in four CRPC cases (#1003, #1005, #1014 and #1060) but not in any of the HSPC cases (Figure 1).

### Plasma genomic abnormality (PGA) score and its clinical association

**Figure 2:**
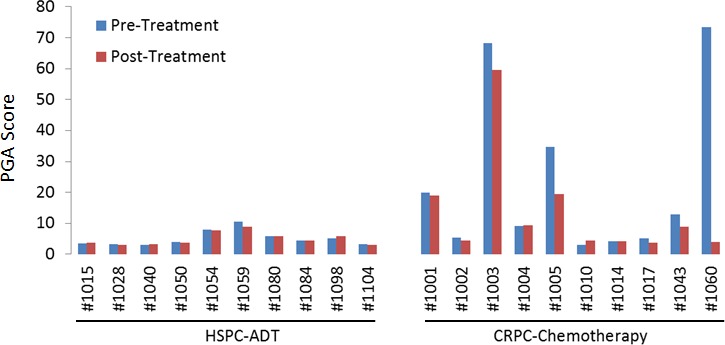
Plasma genomic abnormality (PGA) scores in 20 patients with advanced prostate cancer Higher PGA scores indicated more tumor-associated somatic abnormalities in cfDNA and were associated with disease progression and overall survival.

**Figure 3:**
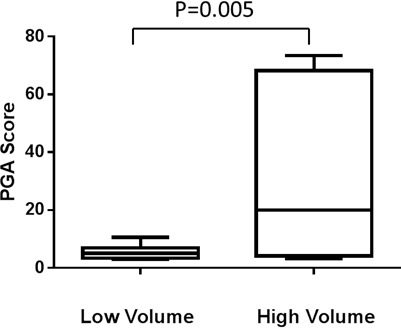
PGA score differences between high and low volume prostate cancer patients (see main text for definition) Average PGA score before treatment is significantly lower in low volume patients (*n* = 13) than in high volume patients (*n* = 7).

To quantify the tumor DNA fraction in cfDNA, we summed the squared 95th-99th absolute log2 ratios as the PGA score. Similar to gross chromosomal abnormality, the PGA scores were significantly higher in the CRPC cohort than in the HSPC cohort (Figure 2). To estimate potential association of PGA scores with overall survival, we performed Cox regression analysis in 19 of the 20 patients with complete follow-up data. We found that elevated PGA scores in pre-treatment samples were significantly associated with short survival (*p* = 0.01, 95% CI = 1.01-1.08). We also observed this association in post-treatment samples (*p* = 0.04, 95% CI = 1.00-1.20). Among the 20 patients, 7 were classified as having high volume disease (Table 1), defined by the presence of either a visceral (non-lymph nodal) metastasis or >4 bone lesions with at least one present outside the spine or pelvis skeleton at the time of initiating chemotherapy for the CRPC stage. Five of the 7 high volume cancer patients showed high initial PGA scores (cutoff value >10) but only 1 of 13 low volume patients demonstrated high initial PGA score (*p* = 0.005, unpaired t test) (Figure 3).

**Table 1:** Clinical characteristics of 20 advanced prostate cancer patients

Patient ID	Age at time of Diagnosis (years)	Patient Group	Treatment	Gleason store at diagnosis	TNM staging at diagnosis	Metastatic status before treatment*	PSA (ng/ml) at time of 1st sample collection in advanced stage	PSA (ng/ml) attime of 2nd sample collection in advanced stage	Time period (days) between two sample collections	Vital status Alive=0; Dead=1	Follow-up time (months)
1001	62	CRPC	Chemo	9	T4N1M1	High Volume	8.2	0.42	147	0	37.12
1002	66	CRPC	Chemo	7	T2cNxM0	Low Volume	9.3	1.6	89	1	17.23
1003	54	CRPC	Chemo	7	T3aN0M0	High Volume	107	162	84	1	644
1004	69	CRPC	Chemo	8	T3aNxM0	Low Volume	3.4	4.6	92	0	49.18
1005	69	CRPC	Chemo	9	T3bN1M0	High Volume	0.48	0.1	146	1	9.07
1010	72	CRPC	Chemo	9	T3bN1M0	High Volume	5	NA	144	1	21.53
1014	61	CRPC	Chemo	7	T2bN1M1	High Volume	126	56.8	99	1	19.82
1017	63	CRPC	Chemo	5	T2aN0M0	Low Volume	22	104	139	0	16.21
1043	73	CRPC	Chemo	7	T2aNxM1	High Volume	15.5	8	80	0	32.22
1060	78	CRPC	Chemo	7	TxNxM1	High Volume	3.7	1.4	104	1	18.48
1015	67	HSPC	ADT	7	T2cNxM0	Low Volume	1	0.9	98	0	53.77
1028	49	HSPC	ADT	9	T3bN0M0	Low Volume	0.33	0.12	154	0	95.27
1040	53	HSPC	ADT	9	T2NxM0	Low Volume	2.5	<0.10	168	0	42.93
1050	64	HSPC	ADT	9	T3bN1M1	Low Volume	4.2	<0.10	136	0	57.47
1054	81	HSPC	ADT	7	T2aNxM0	Low Volume	6A	<0.10	154	1	54.33
1059	62	HSPC	ADT	9	T3bN1M0	Low Volume	2.9	<0.10	116	0	55.20
1080	65	HSPC	ADT	8	T3bN1M0	Low Volume	16	0.77	172	0	49.27
1084	57	HSPC	ADT	9	T3bN0M0	High Volume	2.2	0.24	78	0	53.80
1098	78	HSPC	ADT	6	T2aNxM0	Low Volume	5.7	0.54	131	0	NA
1104	67	HSPC	ADT	9	T2cN1M1	Low Volume	37	<0.10	99	0	51.60

* High Volume Metastatic disease definition: 4 or more metastatic skeletal lesions on hone scan with at least I of the 4 being present outside the pelvic or spinal skeleton and/or presence of visceral metastatic disease (non lymph node disease)

**Figure 4:**
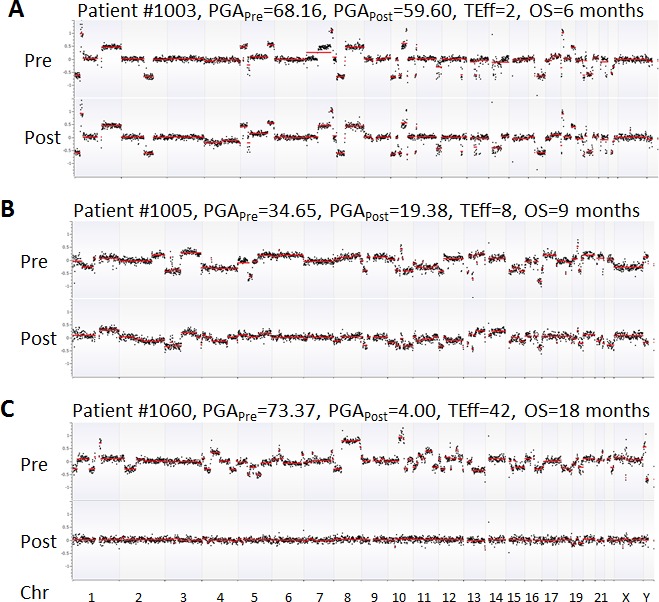
Comparison of PGA scores and TEff indexes in three representative CRPC patients Chromosomes were shown on the x axis while GC-adjusted log2 ratios (black dots) in 1Mb windows were on the y axis. Red lines indicate the trend of copy number variations. Complete, partial and no responses to chemotherapy were displayed in **A** (patient 1060), **B** (patient 1005), and **C** (patient 1003), respectively. OS = overall survival.

For the 10 HSPC patients undergoing ADT, PGA score changes between treatments were minor. This was attributable to relatively low tumor burden in this group of patients. After a median follow-up time of 53.8 months (range 42-95 months), only one patient (#1054) was deceased due to disease. This patient showed relatively high PGA scores in both pre- and post-ADT in the HSPC cohort (Figure 2). For the 10 CRPC patients receiving chemotherapy, the patients with the highest initial PGA score included #1003, 1005 and 1060. All three patients died with relatively short survival time. To estimate patients' response to treatment, we calculated their Treatment Efficacy (TEff) indexes by transforming PGA score differences between pre- and post-treatments (see method section). We found that the TEff indexes in patients 1003, 1005, and 1060 were 2, 8, and 42, respectively. Correspondingly, their overall survival times were 6, 9 and 18 months (Figure 4).

### Cancer gene mutational profiles

To identify somatic mutations in cfDNAs, we performed the targeted sequencing of 578 cancer-related genes in the 20 patients. The average mapped reads per patient was 14.46 million (range 9.11-19.74) with 44% of reads on target (range 41-48%). Sequences of all samples achieved a mean coverage of 79x (range 54-87). Among 10 HSPC patients, we identified somatic mutations in 66 genes in pre-ADT and 68 genes in post-ADT samples after removing constitutional polymorphisms (cfDNA vs. matched gDNA). Of these mutated genes, 17 were shared between pre- and post-treatment samples. Among 10 CRPC patients, we identified somatic mutations in 52 genes in pre-chemotherapy and 63 genes in post-chemotherapy samples, of which 18 genes were shared (Supplementary Tables S1-S4). To validate these mutations, we applied allele-specific PCR (AS-PCR) to examine 26 mutations in 41 samples with mutations found by sequencing technology. AS-PCR successfully confirmed 20 of these mutations (Supplementary Figure S2). The remaining 6 mutations were uncertain due to difficulty in establishing high quality AS-PCR assays.

### Gene mutation profile changes between pre- and post-treatment

**Figure 5:**
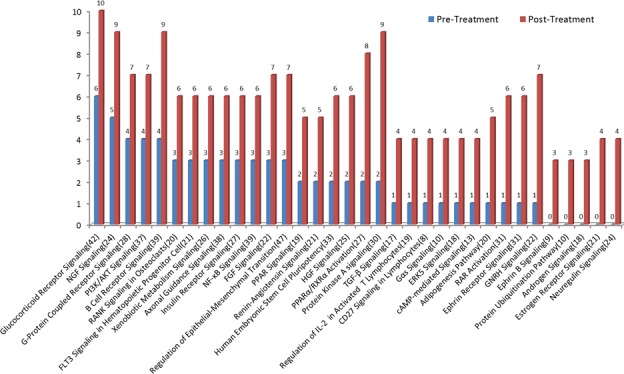
Mutational profile changes between pre-ADT and post-ADT Gene mutation pathway enrichment analysis was performed in the HSPC cohort receiving standard ADT. More gene mutations were observed in post- than in pre-treatment samples. Pathways involved in androgen biosynthesis, metabolism, and androgen receptor activation are among the most commonly mutated.

To examine treatment-associated pathway alterations, we analyzed the two patient cohorts separately. Overall, we observed 34 and 35 pathways showing >3 gene differences between pre- and post-treatment samples in the HSPC and CRPC cohorts, respectively. Compared to pre-treatment samples, mutations in post-treatment samples were more diverse, reflecting more pathways involved. For HSPC patients, we observed more gene mutations in post- than in pre-treatment samples in all pathways (Figure 5). Interestingly, the genes involving androgen biosynthesis and metabolism including androgen signaling, estrogen receptor signaling and GNRH signaling pathways were among the most commonly mutated. For example, GNRH signaling pathway is a target of ADT and contains 22 genes. Of those, only 1 gene mutation was detected before ADT but 7 gene mutations were detected after ADT.

**Figure 6:**
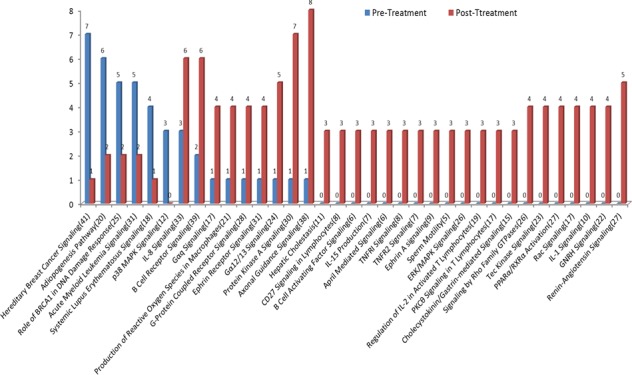
Mutational profile changes between pre- and post-chemotherapy Many mutations detected after chemotherapy were not present in pre-treatment samples. The most common mutations in pre-treatment samples were in DNA repair-related hereditary breast cancer signaling pathways. The most common mutations in post-treatment samples occurred in the pathways related to *AR* regulation and resistance to chemotherapy including axonal guidance signaling, protein kinase A signaling, and renin-angiotensin signaling pathways.

For CRPC patients, 20 of 35 pathways had gene mutations in post-treatment patients only. The most common mutations in the post-treatment group included axonal guidance signaling, protein kinase A signaling and renin-angiotensin signaling pathways. Meanwhile, 6 pathways showed less gene mutations in post- than in pre-treatment samples (Figure 6). The most common mutations before chemotherapy occurred in DNA repair-related hereditary breast cancer signaling genes. Among 41 genes in the pathway, 7 mutations were detected in the pre-treatment but only 1 mutation was found in the post-treatment samples.

## DISCUSSION

Cancer is characterized by massive genomic abnormalities, some of which are targets for therapy or are used for monitoring response to specific treatments. Recent studies have reported that genomic abnormalities in cfDNA resemble genomic signatures of primary tumors in human cancers [3-5, 13]. In this study, we examined plasma cfDNAs in advanced prostate cancer and were able to detect somatic mutations and genomic aberrations in all of the patients after accounting for constitutional genomic abnormalities. These aberrations were often different between pre- and post-treatment, reflecting dynamic genomic evolution during stage-specific therapies. Our results suggest that somatic alterations in cfDNA may serve as sensitive biomarkers for predicting treatment response and clinical outcome in advanced prostate cancer.

To examine the repertoire of genomic aberrations in tumor tissues, biopsies are often performed. However, tissue biopsy in advanced prostate cancer is challenging because bone metastasis are predominant. Many patients do not have residual disease at their primary site due to surgical removal of the prostate. Biopsies at sites of bone or nodal metastasis are invasive, morbid, and inaccurate. These biopsies are subject to sampling bias and may not represent the overall tumor mass. Due to these limitations, liquid biopsy by sensitive detection of tumor components has emerged as an attractive alternative option. This approach is minimally invasive and can be more frequently scheduled in clinical laboratories. Because blood stream contains the cfDNAs derived from all tumor sites, the liquid biopsy assay may detect more complete repertoire of tumor genome variations [3-5, 10, 13]. It has been shown that tumor genomic abnormalities are well reflected in cfDNA during cancer progression [7, 8]. By comparing the differences between multiregional sequencing of 2 synchronous cancer tissues and shotgun sequencing of cfDNA, Chan et al show that cfDNA sequencing is able to detect genomic variations originated from different tumor sites [10]. Recently, Schutz et al found that cfDNA genomic variations are able to distinguish both benign prostatic hypertrophy and prostatitis from prostate cancer with accuracy of 90% [17]. Clearly, liquid biopsy may provide a useful tool for cancer detection, monitoring and research.

To estimate tumor DNA content, previous studies applied “genomewide z-score” [7] or “PA-score” [8]. However, these algorithms may not accurately reflect tumor DNA contribution to cfDNA because tumor genomes are not always altered in all genome segments. In addition, calculations of these scores require cfDNAs derived from a group of normal individuals as reference controls. Due to the germline-determined CNVs pre-existing in any given individual, these algorithms may generate significant bias toward the regions with pre-existing CNVs. To address this issue, we normalized cfDNA read counts using lymphcyte gDNA read counts from the same patient, significantly minimizing the biases caused by pre-existing CNVs. Additionally, we developed the PGA scoring system by summing the most significant genomic regions, avoiding potential background noises from other scoring algorithms. Our data show that PGA scores and TEff indexes are potentially useful to assess treatment response and overall survival.

Targeted sequencing in cfDNA has demonstrated potential clinical utility in guiding selection of targeted therapies [26]. By analyzing mutational profiles before and after initiating ADT, we were able to detect increased mutant genes after ~4 months of ADT in several critical pathways, including protein kinase A signaling, the PPARα/RXRα activation and GNRH signaling pathways. These pathways are associated with *AR* activation [27] and androgen biosynthesis [28]. One key mutated gene in these pathways is *EP300*, a crucial gene for prostate cancer cell proliferation [29] and hormone responsiveness of *AR* [30]. We also found more gene mutations in the glucocorticoid receptor (*GR*) signaling pathway after ADT. *GR* expression is stimulated by castration therapy, a mechanism that compensates for *AR* signaling blockade and promotes CRPC progression [31, 32]. Currently, preclinical models are often used to define the mechanisms of resistance to a specific treatment, but it is generally difficult to confirm these findings in clinical samples. Our results suggest that cfDNA-based genetic analysis may provide a powerful and easily accessible approach for studying tumor resistance in real patient samples.

Many mutations detected after treatments were not present in pre-treatment samples. These non-overlapping mutations are of interest as they may provide novel insights into the evolution of tumor genomes in response to therapy or serve as predictive biomarker for treatment response and/or prognostic biomarkers for survival. For example, *PRKAR1A* and *NFKB2* were found to be mutated after chemotherapy. *PRKAR1A* is functionally linked to *AR* during the progression of prostate cancer [33]. Its overexpression is observed in advanced prostate cancer [33, 34] and may cause resistance to chemotherapy [35]. *NFKB* can be activated by the chemotherapy drug (docetaxel) and contributes to treatment resistance in prostate cancer [36-38]. These results are consistent with the common notion that stage-specific therapies increase tumor cell subpopulations carrying treatment-resistant mutations and proportionally reduce cell subpopulations carrying treatment-sensitive mutations.

In summary, we applied next generation sequencing to test cfDNAs for somatic variations in advanced prostate cancer. We developed a new scoring algorithm to estimate tumor DNA burden and predict patient's response to a specific therapy. We found that genetic and genomic profile changes after treatments are clinically and biologically associated with response to stage-specific therapies. Although the study examined a limited number of patients, the results from this study strongly support that DNA-based liquid biopsy has great potential to serve as alternative means to examine tumor genetic changes in advanced prostate cancer. Further studies are needed to justify the clinical utility of cfDNA as useful biomarker to predict treatment response and clinical outcomes.

## MATERIALS AND METHODS

### Sample collection

Plasma specimens from two separate cohorts of advanced prostate cancer patients were randomly selected from a hospital-based registry for biomarker development in advanced prostate cancer. Details of patient enrollment have been previously reported [39]. The plasma was derived from EDTA-treated blood. All plasma was separated within 2 hours after blood draw and frozen immediately at −80ÐC without any freeze-thaw cycle before use. Patient characteristics are presented in Table 1. Each patient provided plasma collected just before treatment and plasma collected approximately four months after initiating stage-specific therapy. The treatments were initiated after collection of the first specimen. Castration levels of testosterone (total testosterone < 50ng/dl) were confirmed at the time of the second sample collection. This study was approved by Institutional Review Boards at both the Medical College of Wisconsin and Mayo Clinic.

### DNA extraction and sequencing library preparation

Blood plasma samples underwent a second centrifugation at 3000 rpm for 10 min before DNA extraction. The cfDNAs were extracted from 400-800μl of plasma using QIAamp DNA Blood Mini Kit (QIAGEN, Valencia, CA, USA). The final DNA eluent (50μl) was quantified by a Qubit 2.0 Fluorometer (Life Technology, Carlsbad, CA, USA) and stored at −80°C until use. DNA libraries were prepared using a NEXTflex DNA-Seq Kit (BIOO Scientific Corporation, Austin, TX, USA). Libraries were pooled for paired-end sequencing on a HiSeq2000 Sequencing System (Illumina, San Diego, CA, USA).

### CNV calculation

Raw sequencing data (fastq files) were first mapped to the human genome (hg19) (DNASTAR, Madison, WI). Read counts from the mapped sequence files were then binned into 1Mb windows (total 3113 genomic bins) and adjusted to the global mean count for each sample. The read count ratio in each genomic bin was calculated by comparing cfDNA to lymphocyte gDNA derived from the same patient to account for constitutional CNVs. The resulting ratios were further log2 transformed and corrected for GC content [40]. The fully normalized log2 ratios in genomic bins were subjected to segmentation using the copy number analysis method (CNAM) algorithm (Golden Helix, Bozeman, MT).

### PGA score and TEff index

To quantify the genomic abnormality and facilitate comparison between different samples, we defined the *i*th percentile of absolute log2 ratios (ALRs) as ALR.*i* and calculated the sum of all squared ALRs between ALR.95 and ALR.99, where ALR.95 was considered as the minimum threshold of genomic abnormality. We named this summed value “Plasma Genomic Abnormality (PGA) score”. A higher score indicates greater tumor DNA fraction in the cfDNA. The top one percentile ALRs were excluded to avoid over-estimation of genomic abnormalities because some samples showed extensive CNVs at telomere or centromere regions (Supplementary Figure S3). Although we couldn't exclude possibility of true CNV changes (for example, gene amplification), we believe that the extreme CNV changes in the regions were more likely caused by high sequence homologs and relatively low quality sequencing libraries. To quantify treatment response in each patient, we defined the TEff (Treatment Efficacy) index as the log2 ratio of PGA scores between the pre- and post-treatments: TEff index=log2(prePGA/postPGA) x10. A TEff index of less than or close to zero indicates no response to treatment while a higher TEff index is indicative of a better treatment response.

### Targeted sequencing

The Comprehensive Cancer Panel (Roche NimbleGen, Madison, WI) was used for targeted sequencing. The panel covers 4Mb genomic sequences and targets 578 cancer-related genes. The genes were captured from sequencing libraries made for CNV analysis according to Roche NimbleGen's manual. Final enriched libraries were subjected to 100bp PE sequencing on a HiSeq2000 Sequencing System. Gene mutations were detected by comparing cfDNA to lymphocyte gDNA in the same patient with 2% variant alleles as the cutoff for mutation calls.

### Allele specific PCR

Allele specific PCR (AS-PCR) was used to validate sequencing-detected mutations. For each mutation, three primers were designed with one common primer and two mutant-specific primers. Reactions were performed in a 25μl reaction with 4ng of pre-amplified DNA and 0.5 unit of Taq DNA polymerase (New England Biolab, Ipswich, MA). This DNA polymerase does not have 3′-5′ exonuclease activity and therefore is suitable for AS-PCR. Amplifications were carried out in a thermal cycler (Eppendorf Mastercycler pro S) including initial denaturation for 60sec at 95°C, 40 cycles of denaturation for 30sec at 95°C, annealing for 30sec at primer-dependent temperatures (Supplementary Table S5), and extension for 40sec at 72°C.

### Mutation pathway enrichment analysis

To examine the functional classifications of mutant genes, we applied Ingenuity Pathway Analysis (IPA, Qiagen, CA) and treated the 578 cancer-related genes as background reference. For mutant genes, we searched for mutational profile differences between pre- and post-treatment samples. We defined >3 gene differences in a specific pathway between pre- and post-treatments as the cutoff for mutational profile changes. This analysis was useful to determine critical pathways in response to stage-specific therapy.

## SUPPLEMENTARY MATERIAL FIGURES AND TABLES


