# Supplementary material for: Plasma genetic and genomic abnormalities predict treatment response and clinical outcome in advanced prostate cancer
Source: Oncotarget. 2015 Apr 15;6(18):16411–21. doi: 10.18632/oncotarget.3845 (PMC4599278; doi:10.18632/oncotarget.3845)
Supplement: Supplementary file 1 [file oncotarget-06-16411-s001.pdf]

Plasma genetic and genomic abnormalities predict treatment response and clinical outcome in advanced prostate cancer

Supplementary Material

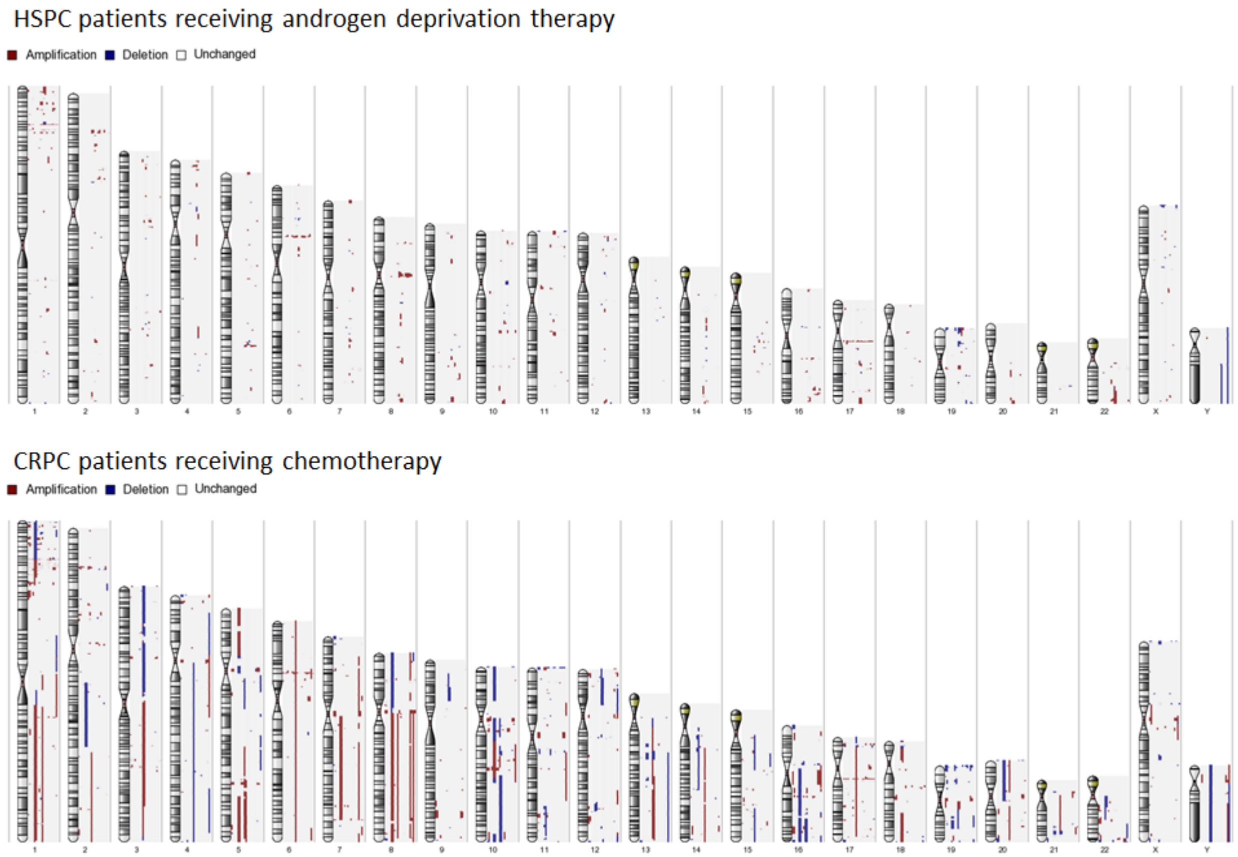

Supplementary Figure S1: Overall copy number variations in cfDNAs derived from advanced prostate cancer patients. Copy number variations determined by log2 ratios between cfDNA and matched gDNA were shown in chromosomes 1 through X and Y. Red bars represent chromosome segment amplification (log2 ratios ≥0.2) while blue bars represent segment deletion (log2 ratios ≤-0.2). There were more copy number changes in CRPC (lower panel) than in HSPC (upper panel) patients.

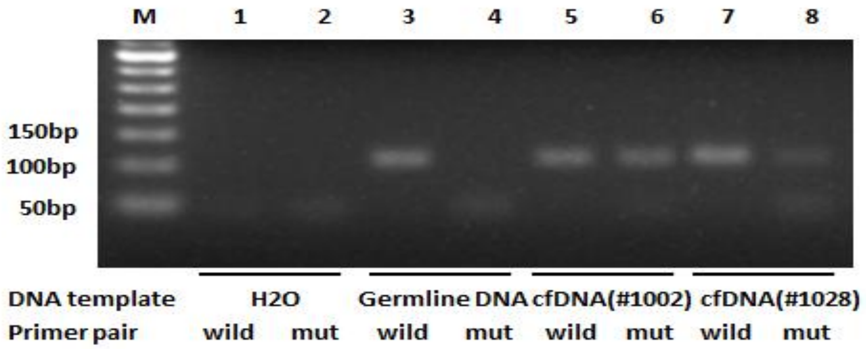

Supplementary Figure S2: Representative AS-PCR for mutation validation. 103bp fragments in *NUP214* were amplified by AS-PCR and subjected to agarose gel electrophoresis. Lanes 1, 3, 5 and 7 were wildtype-specific primer pairs. Lane 2, 4, 6 and 8 are mutant-specific primer pairs. Lanes 1 and 2 were blank control without DNA template. Lanes 3 and 4 were wildtype genomic DNA. Lanes 5 and 6 were cfDNAs from patient #1002. Lanes 7 and 8 were cfDNAs from patient #1028. Mutants were detected in lanes 6 and 8.

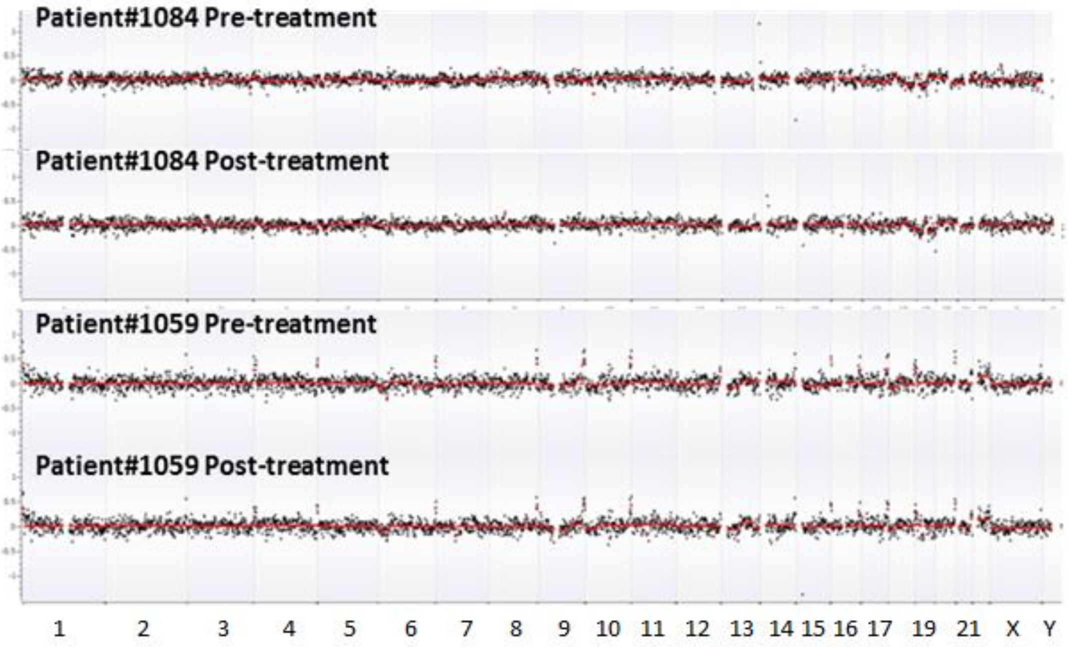

Supplementary Figure S3: Extensive CNVs at telomere and centromere regions in patient #1059. Chromosomes were shown on the x axis while GC-adjusted log2 ratios (black dots) in 1Mb windows were on the y axis. Red lines indicate the trend of copy number variations. Extensive CNV at telomere and centromere regions were clearly seen in patient #1059 but not in patient #1084.

Supplementary Table S1 - Gene mutations in pre-treatment patients only

Chromosome	Position	Patient ID	Treatment	Mutant Allele Frequency % Pre-Treatment	Depth	Gene name	DNA Change	Amino Acid Change
chr10	81465798	1043	Chemotherapy	25.9	54	NUTM2B	c.383C>T	p.A128V
chr02	48040930	1001	Chemotherapy	7.9	164	FBXO11	c.2083G>A	p.G695S
chr01	27101460	1010	Chemotherapy	7.8	64	ARID1A	c.4742A>C	p.H1581P
chr01	11199588	1043	Chemotherapy	7.2	69	MTOR	c.5000T>G	p.V1667G
chr10	114711242	1002	Chemotherapy	5.3	130	TCF7L2	c.257C>T	p.A86V
chr20	39742611	1005	Chemotherapy	4.9	101	TOP1	c.1454T>C	p.L485P
chr14	105239429	1017	Chemotherapy	4.9	141	AKT1	c.958G>A	p.V320M
chr14	56145161	1002	Chemotherapy	4.8	124	KTN1	c.3947C>T	p.T1316M
chr01	157550138	1060	Chemotherapy	4.8	83	FCRL4	c.1250G>T	p.G417V
chr09	134106018	1017	Chemotherapy	4.1	97	NUP214	c.6202T>G	p.F2068V
chr05	149437068	1002	Chemotherapy	3.8	129	CSF1R	c.2220A>T	p.Q740H
chr17	8050789	1002	Chemotherapy	3.5	57	PER1	c.1496A>C	p.Q499P
chr13	29041658	1005	Chemotherapy	3.5	170	FLT1	c.161G>T	p.R54M
chr14	105239428	1005	Chemotherapy	3.4	144	AKT1	c.959T>G	p.V320G
chr01	16248745	1001	Chemotherapy	3.3	219	SPEN	c.1751T>G	p.V584G
chr23	44833911	1003	Chemotherapy	3.3	59	KDM6A	c.335C>A	p.A112E
chr09	134015937	1002	Chemotherapy	3.3	121	NUP214	c.1257T>G	p.S419R
chr02	113984674	1005	Chemotherapy	3.2	153	PAX8	c.1339T>G	p.W447G
chr04	153271195	1001	Chemotherapy	3.2	91	FBXW7	c.583A>G	p.S195G
chr03	37053312	1003	Chemotherapy	3.1	129	MLH1	c.547T>A	p.Y183N
chr11	117096648	1010	Chemotherapy	3.1	96	PCSK7	c.859A>G	p.S287G
chr01	164818578	1003	Chemotherapy	3.1	189	PBX1	c.1202A>C	p.H401P
chr05	176715820	1003	Chemotherapy	3.1	95	NSD1	c.6152G>T	p.G2051V

chr17	17124847	1003	Chemotherapy	3.0	66	FLCN	c.875A>G	p.E292G
chr13	28979918	1002	Chemotherapy	3.0	164	FLT1	c.1550A>G	p.K517R
chr16	89849267	1003	Chemotherapy	2.9	203	FANCA	c.1626G>T	p.E542D
chr06	168291542	1001	Chemotherapy	2.9	302	MLLT4	c.1007G>T	p.G336V
chr05	170827158	1001	Chemotherapy	2.8	321	NPM1	c.526delG	p.M176fs
chro5	170827159	1001	Chemotherapy	2.8	321	NPM1	c.527delA	p.V175fs
chr02	216191545	1005	Chemotherapy	2.8	211	ATIC	c.532G>A	p.A178T
chr01	154143187	1043	Chemotherapy	2.7	72	TPM3	c.643T>A	p.Y215N
chr11	71735321	1010	Chemotherapy	2.6	113	NUMA1	c.207G>T	p.Q69H
chr17	8053751	1003	Chemotherapy	2.5	78	PER1	c.274A>T	p.S92C
chr19	11130267	1010	Chemotherapy	2.5	159	SMARCA4	c.2506G>T	p.G836.
chr19	16192724	1010	Chemotherapy	2.5	118	TPM4	c.242C>T	p.A81V
chr06	33290639	1005	Chemotherapy	2.5	78	DAXX	c.53G>T	p.S18I
chr20	40141486	1001	Chemotherapy	2.5	347	CHD6	c.851A>G	p.E284G
chr15	74326873	1005	Chemotherapy	2.5	118	PML	c.1712T>G	p.V571G
chr15	74327477	1010	Chemotherapy	2.5	80	PML	c.1715C>T	p.S572F
chr06	117704481	1017	Chemotherapy	2.5	198	ROS1	c.2495delA	p.K832fs
chr02	128017023	1010	Chemotherapy	2.5	159	ERCC3	c.2066T>G	p.V689G
chr17	7579508	1003	Chemotherapy	2.4	82	TP53	c.179C>A	p.P60Q
chr07	26233195	1010	Chemotherapy	2.4	122	HNRNPA2B1	c.877G>T	p.G293.
chr16	10989527	1043	Chemotherapy	2.3	347	CIITA	c.246A>C	p.E82D
chr06	168289896	1002	Chemotherapy	2.3	129	MLLT4	c.896T>A	p.V299D
chr17	7578553	1001	Chemotherapy	2.2	226	TP53	c.377A>C	p.Y126S
chr19	7705606	1017	Chemotherapy	2.2	134	STXBP2	c.269A>C	p.H90P
chr17	29557279	1002	Chemotherapy	2.2	87	NF1	c.2992T>C	p.Y998H
chr08	38274934	1010	Chemotherapy	2.2	136	FGFR1	c.1646C>A	p.S549.
chr11	119155899	1005	Chemotherapy	2.2	88	CBL	c.1564G>T	p.A522S
chr04	1932354	1005	Chemotherapy	2.1	140	WHSC1	c.1412T>G	p.V471G
chr22	40831504	1060	Chemotherapy	2.1	189	MKL1	c.167G>A	p.R56K
chr12	57911053	1010	Chemotherapy	2.1	139	DDIT3	c.204_206delAGA	p.E68_E69delinsE

chr01	92752047	1060	Chemotherapy	2.1	93	GLMN	c.735A>G	p.I245M
chr06	167453392	1010	Chemotherapy	2.1	94	FGFR1OP	c.1126C>T	p.L376F
chr02	25523009	1010	Chemotherapy	2.0	143	DNMT3A	c.176delC	p.P59fs
chr22	29120965	1060	Chemotherapy	2.0	148	CHEK2	c.721G>A	p.V241I
chr23	70320535	1059	ADT	7.9	50	FOXO4	c.455A>C	p.N152T
chr03	178951883	1028	ADT	6.4	78	PIK3CA	c.2938T>G	p.F980V
chr05	131325794	1080	ADT	6.2	96	ACSL6	c.449A>G	p.E150G
chr08	134271412	1080	ADT	6.0	116	NDRG1	c.388G>T	p.G130W
chr19	1220373	1028	ADT	5.3	56	STK11	c.466T>G	p.Y156D
chr16	15851685	1028	ADT	5.2	57	MYH11	c.1595C>T	p.P532L
chr08	90949302	1084	ADT	5.1	78	NBN	c.2186T>A	p.V729E
chr23	133547521	1015	ADT	4.7	87	PHF6	c.422C>A	p.A141D
chr10	102896426	1104	ADT	4.6	65	TLX1	c.772T>G	p..258E
chr02	25505304	1104	ADT	4.4	89	DNMT3A	c.454T>G	p.S152A
chr03	188426055	1028	ADT	4.4	89	LPP	c.1204G>T	p.G402C
chr17	17127456	1028	ADT	4.0	98	FLCN	c.398T>G	p.V133G
chr09	139413043	1028	ADT	3.9	51	NOTCH1	c.1099G>A	p.G367S
chr02	223066160	1028	ADT	3.8	103	PAX3	c.1422T>G	p.S474R
chr17	5286418	1028	ADT	3.7	80	RABEP1	c.2489T>G	p.V830G
chr02	100721967	1080	ADT	3.5	84	AFF3	c.322C>T	p.R108C
chr09	134015937	1028	ADT	3.4	88	NUP214	c.1257T>G	p.S419R
chr09	135985684	1050	ADT	3.4	88	RALGDS	c.487A>G	p.R163G
chr24	15470400	1098	ADT	3.2	61	UTY	c.1420C>A	p.Q474K
chr03	142168444	1084	ADT	3.2	62	ATR	c.7762G>A	p.A2588T
chr10	63816877	1084	ADT	3.1	159	ARID5B	c.848T>G	p.V283G
chr15	91185167	1104	ADT	3.1	96	CRTC3	c.1655A>C	p.D552A
chr11	108205697	1104	ADT	3.1	129	ATM	c.8012T>G	p.V2671G
chr11	106856796	1028	ADT	3.0	132	GUCY1A2	c.365G>T	p.G122V
chr01	206669446	1028	ADT	3.0	99	IKBKE	c.2119C>A	p.L707I
chr01	2489166	1104	ADT	2.9	103	TNFRSF14	c.71T>G	p.V24G

chr06	28872442	1015	ADT	2.9	300	TRIM27	c.947T>A	p.V316E
chr19	30311610	1015	ADT	2.9	100	CCNE1	c.464T>G	p.V155G
chr02	190670379	1015	ADT	2.9	101	PMS1	c.317T>A	p.V106D
chr11	67257510	1104	ADT	2.8	107	AIP	c.470T>G	p.V157G
chr11	68177382	1028	ADT	2.8	173	LRP5	c.2092A>C	p.T698P
chr11	118363772	1015	ADT	2.8	107	KMT2A	c.5005G>A	p.A1669T
chr11	128846306	1028	ADT	2.8	173	LRP5	c.2092A>C	p.T698P
chr09	132686221	1098	ADT	2.8	141	FNBP1	c.1042delC	p.Q348fs
chr17	37054666	1015	ADT	2.7	146	LASP1	c.251T>G	p.V84G
chr10	70442594	1098	ADT	2.7	185	TET1	c.4916T>G	p.V1639G
chr16	89805887	1015	ADT	2.7	110	FANCA	c.4009delA	p.S1337fs
chr15	99500291	1015	ADT	2.7	109	IGF1R	c.3724T>G	p.F1242V
chr02	208442312	1084	ADT	2.7	182	CREB1	c.814C>T	p.P272S
chr14	68758602	1084	ADT	2.6	151	RAD51B	c.758T>G	p.V253G
chr02	97215059	1028	ADT	2.6	75	ARID5A	c.122A>C	p.D41A
chr15	99500291	1104	ADT	2.6	150	IGF1R	c.3724T>G	p.F1242V
chr01	3348531	1050	ADT	2.5	116	PRDM16	c.3526T>G	p.C1176G
chr19	4364128	1104	ADT	2.5	118	SH3GL1	c.422A>C	p.D141A
chr12	4398155	1028	ADT	2.5	118	CCND2	c.719T>G	p.V240G
chr03	52584764	1080	ADT	2.5	238	PBRM1	c.4778A>C	p.Q1593P
chr15	74883901	1098	ADT	2.5	78	ARID3B	c.1166G>T	p.G389V
chr19	45297462	1028	ADT	2.4	124	CBLC	c.1286T>G	p.V429G
chr09	123933826	1050	ADT	2.4	121	CNTRL	c.6417G>T	p.Q2139H
chr17	29685988	1040	ADT	2.3	84	NF1	c.8115delT	p.S2705fs
chr10	43620332	1028	ADT	2.3	127	RET	c.2941T>G	p.Y981D
chr03	186502485	1028	ADT	2.3	85	EIF4A2	c.211G>T	p.G71W
chr01	3348531	1104	ADT	2.2	218	PRDM16	c.3526T>G	p.C1176G
chr17	9862581	1028	ADT	2.2	131	GAS7	c.43T>C	p.S15P
chr14	51196242	1040	ADT	2.2	135	NIN	c.6077A>G	p.Q2026R
chr06	135511266	1015	ADT	2.2	175	MYB	c.308T>G	p.V103G
chr02	25470026	1050	ADT	2.1	182	DNMT3A	c.1016T>G	p.V339G
chr19	45916933	1080	ADT	2.1	137	ERCC1	c.845T>A	p.V282E
chr09	98211605	1040	ADT	2.1	93	PTCH1	c.3550G>A	p.V1184M
chr13	103518017	1040	ADT	2.1	91	ERCC5	c.1955G>T	p.G652V
chr13	103518017	1040	ADT	2.1	91	BIVM-ERCC5	c.3317G>T	p.G1106V
chr08	134274395	1104	ADT	2.1	322	NDRG1	c.221A>C	p.N74T
chr04	1932354	1028	ADT	2.0	197	WHSC1	c.1412T>G	p.V471G
chr17	30315340	1054	ADT	2.0	146	SUZ12	c.1025G>T	p.R342M
chr17	36881810	1104	ADT	2.0	143	MLLT6	c.3322A>T	p.T1108S
chr11	68177382	1098	ADT	2.0	147	LRP5	c.2092A>C	p.T698P
chr17	79941431	1050	ADT	2.0	149	ASPSCR1	c.160T>G	p.F54V
chr11	125514408	1028	ADT	2.0	146	CHEK1	c.1103A>C	p.N368T

Supplementary Table S2 - Gene mutations in post-treatment patients only

Chromosome	Position	Patient ID	Treatment	Mutant Allele Frequency % Post-Treatment	Depth	Gene name	DNA Change	Amino Acid Change
chr23	44918252	1001	Chemotherapy	15.6	51	KDM6A	c.877T>G	p.C293G
chr01	11199588	1005	Chemotherapy	8.3	84	MTOR	c.5000T>G	p.V1667G
chr17	5036205	1001	Chemotherapy	6.9	472	USP6	c.196A>C	p.K66Q
chr17	39778604	1005	Chemotherapy	6.5	55	KRT17	c.673_675delGTG	p.V225del
chr01	11199588	1001	Chemotherapy	6.5	138	MTOR	c.5000T>G	p.V1667G
chr02	1.28E+08	1017	Chemotherapy	5.8	102	ERCC3	c.2066T>G	p.V689G
chr19	18856633	1017	Chemotherapy	5.5	90	CRTC1	c.292A>C	p.T98P
chr19	18856633	1001	Chemotherapy	5.5	179	CRTC1	c.292A>C	p.T98P
chr08	1.42E+08	1002	Chemotherapy	5.5	217	PTK2	c.2478_2483delTTACCA	p.H826_Q828delinsQ
chr09	21974675	1017	Chemotherapy	5.3	56	CDKN2A	c.152T>G	p.V51G
chr05	1.12E+08	1017	Chemotherapy	5.0	79	APC	c.935T>G	p.V312G
chr01	1.65E+08	1001	Chemotherapy	5.0	179	PBX1	c.1202A>C	p.H401P
chr10	88988020	1001	Chemotherapy	4.9	101	NUTM2A	c.383C>T	p.A128V
chr10	43622023	1005	Chemotherapy	4.4	89	RET	c.3040G>T	p.D1014Y
chr09	21974675	1005	Chemotherapy	4.3	91	CDKN2A	c.152T>G	p.V51G
chr17	66523982	1005	Chemotherapy	4.2	165	PRKAR1A	c.710G>T	p.G237V
chr09	98268880	1014	Chemotherapy	4.2	94	PTCH1	c.203G>C	p.G68A
chr06	1.18E+08	1017	Chemotherapy	4.1	193	ROS1	c.5777A>G	p.H1926R
chr01	1.57E+08	1043	Chemotherapy	3.8	77	NTRK1	c.2117T>G	p.V706G
chr14	56107845	1001	Chemotherapy	3.7	107	KTN1	c.2022T>G	p.S674R
chr22	24143268	1017	Chemotherapy	3.6	222	SMARCB1	c.473G>T	p.W158L
chr03	47088110	1005	Chemotherapy	3.6	111	SETD2	c.6964_6965insT	p.S2322fs
chr08	1.42E+08	1017	Chemotherapy	3.5	57	PTK2	c.662G>T	p.R221L
chr03	47088111	1005	Chemotherapy	3.5	113	SETD2	c.6964A>T	p.S2322C

chr05	1.77E+08	1002	Chemotherapy	3.4	176	NSD1	c.5147G>A	p.G1716E
chr07	1.43E+08	1003	Chemotherapy	3.2	91	EPHB6	c.2959G>A	p.D987N
chr01	1.86E+08	1060	Chemotherapy	3.2	124	TPR	c.6796G>T	p.G2266C
chr17	12920437	1043	Chemotherapy	3.1	95	ELAC2	c.247T>G	p.Y83D
chr16	23693386	1001	Chemotherapy	3.1	352	PLK1	c.724T>G	p.Y242D
chr03	52620442	1003	Chemotherapy	3.1	129	PBRM1	c.3485delA	p.K1162fs
chr01	2.07E+08	1017	Chemotherapy	3.1	64	IKBKE	c.1342G>A	p.E448K
chr23	70354208	1043	Chemotherapy	3.0	98	MED12	c.4619T>G	p.V1540G
chr14	56105903	1017	Chemotherapy	3.0	98	KTN1	c.1786A>C	p.T596P
chr05	56184054	1017	Chemotherapy	3.0	65	MAP3K1	c.4259T>A	p.V1420E
chr23	70354208	1010	Chemotherapy	2.9	134	MED12	c.4619T>G	p.V1540G
chr07	91674323	1014	Chemotherapy	2.9	101	AKAP9	c.5260T>A	p.Y1754N
chr11	65836145	1005	Chemotherapy	2.8	107	SF3B2	c.2617_2618insA	p.Q873fs
chr09	14146688	1002	Chemotherapy	2.8	138	NFIB	c.925G>A	p.D309N
chr02	25470026	1010	Chemotherapy	2.8	177	DNMT3A	c.1016T>G	p.V339G
chr19	7705606	1014	Chemotherapy	2.7	108	STXBP2	c.269A>T	p.H90L
chr19	45296732	1017	Chemotherapy	2.7	144	CBLC	c.1139A>C	p.H380P
chr10	1.04E+08	1001	Chemotherapy	2.7	71	NFKB2	c.2073_2084delTGCTGACATCCA	p.G691_H695delinsG
chr08	57128992	1060	Chemotherapy	2.7	111	CHCHD7	c.130G>T	p.E44.
chr01	10434375	1001	Chemotherapy	2.7	220	KIF1B	c.4948A>C	p.T1650P
chr09	1.34E+08	1003	Chemotherapy	2.6	149	NUP214	c.1894T>G	p.F632V
chr09	1.39E+08	1017	Chemotherapy	2.6	76	NOTCH1	c.2352C>G	p.S784R
chr01	19062126	1043	Chemotherapy	2.6	75	PAX7	c.1156G>T	p.V386L
chr01	1.65E+08	1014	Chemotherapy	2.6	153	PBX1	c.1202A>C	p.H401P
chr22	31724773	1005	Chemotherapy	2.5	117	PATZ1	c.1645G>T	p.E549.
chr16	16269766	1043	Chemotherapy	2.5	120	ABCC6	c.2326T>G	p..776G
chr16	23693386	1043	Chemotherapy	2.5	240	PLK1	c.724T>G	p.Y242D
chr10	1.03E+08	1043	Chemotherapy	2.4	124	PAX2	c.307T>G	p.Y103D
chr07	1.43E+08	1017	Chemotherapy	2.4	123	EPHB6	c.101A>T	p.E34V
chr04	1941510	1060	Chemotherapy	2.4	82	WHSC1	c.1886_1888delAAT	p.K629_630delinsK
chr02	1.28E+08	1001	Chemotherapy	2.4	250	ERCC3	c.2066T>G	p.V689G

chr10	1.04E+08	1010	Chemotherapy	2.3	127	NFKB2	c.2225T>C	p.V742A
chr08	48846525	1010	Chemotherapy	2.3	211	PRKDC	c.1621_1623delATG	p.M541del
chr07	1.43E+08	1003	Chemotherapy	2.3	126	EPHB6	c.101A>G	p.E34G
chr11	1.17E+08	1003	Chemotherapy	2.2	90	PCSK7	c.914A>C	p.K305T
chr09	1.33E+08	1003	Chemotherapy	2.2	132	FNBP1	c.1036G>T	p.G346C
chr06	1.07E+08	1017	Chemotherapy	2.2	132	PRDM1	c.1775T>G	p.V592G
chr03	97367131	1014	Chemotherapy	2.2	90	EPHA6	c.1154A>C	p.D385A
chr03	1.86E+08	1014	Chemotherapy	2.2	89	ETV5	c.495C>A	p.N165K
chr17	8050569	1017	Chemotherapy	2.1	94	PER1	c.1628C>A	p.P543Q
chr17	79941431	1017	Chemotherapy	2.1	139	ASPSCR1	c.160T>G	p.F54V
chr16	16269766	1060	Chemotherapy	2.1	188	ABCC6	c.2326T>G	p..776G
chr09	35076430	1060	Chemotherapy	2.1	139	FANCG	c.1075A>G	p.R359G
chr03	48719479	1005	Chemotherapy	2.1	93	NCKIPSD	c.597T>A	p.S199R
chr01	38188713	1010	Chemotherapy	2.1	95	EPHA10	c.1960G>A	p.G654R
chr23	44969325	1003	Chemotherapy	2.0	99	KDM6A	c.4163T>G	p.V1388G
chr22	40831504	1010	Chemotherapy	2.0	243	MKL1	c.167G>T	p.R56M
chr09	87563377	1017	Chemotherapy	2.0	198	NTRK2	c.1765A>C	p.T589P
chr08	27303312	1017	Chemotherapy	2.0	288	PTK2B	c.2216T>G	p.V739G
chr04	55968064	1014	Chemotherapy	2.0	146	KDR	c.2266G>T	p.G756C
chr01	1.45E+08	1017	Chemotherapy	2.0	199	PDE4DIP	c.3490T>G	p.C1164G
chr19	4365568	1084	ADT	8.1	74	SH3GL1	c.242T>G	p.V81G
chr17	39778607	1028	ADT	7.7	51	KRT17	c.672_673delAG	p.224fs
chr22	41531818	1028	ADT	7.0	128	EP300	c.1530T>G	p.S510R
chr08	1.42E+08	1080	ADT	6.3	141	PTK2	c.326G>A	p.R109K
chr01	3322060	1028	ADT	5.9	50	PRDM16	c.1037T>G	p.V346G
chr05	1.5E+08	1028	ADT	5.5	90	PDGFRB	c.40G>A	p.G14S
chr12	1.13E+08	1084	ADT	5.1	58	PTPN11	c.1381T>G	p..461G
chr09	1.24E+08	1080	ADT	4.8	104	CNTRL	c.1652C>T	p.S551F
chr05	1.77E+08	1040	ADT	4.6	65	NSD1	c.4303T>C	p.C1435R
chr19	7703906	1028	ADT	4.5	66	STXBP2	c.89T>G	p.V30G
chr06	18258586	1104	ADT	4.4	67	DEK	c.196T>G	p.L66V

chr19	16186858	1104	ADT	4.2	95	TPM4	c.116T>G	p.V39G
chr11	65836146	1098	ADT	4.2	70	SF3B2	c.2618delA	p.Q873fs
chr06	41654832	1080	ADT	4.2	94	TFEB	c.845T>C	p.L282P
chr01	10434375	1040	ADT	3.7	134	KIF1B	c.4948A>C	p.T1650P
chr03	52441975	1028	ADT	3.6	82	BAP1	c.374A>G	p.E125G
chr02	2.08E+08	1084	ADT	3.6	55	CREB1	c.304A>C	p.I102L
chr11	1.19E+08	1080	ADT	3.5	141	CBL	c.2038C>A	p.P680T
chr23	70354208	1098	ADT	3.4	88	MED12	c.4619T>G	p.V1540G
chr06	44220782	1050	ADT	3.4	86	HSP90AB1	c.1732G>T	p.V578L
chr23	70357576	1054	ADT	3.3	59	MED12	c.5836G>T	p.G1946C
chr11	1.19E+08	1084	ADT	3.2	124	CBL	c.197T>G	p.V66G
chr03	1.29E+08	1028	ADT	3.2	123	CNBP	c.223G>A	p.A75T
chr01	18960797	1080	ADT	3.2	93	PAX7	c.86T>G	p.V29G
chr12	1.12E+08	1054	ADT	3.1	96	ALDH2	c.1523T>G	p.V508G
chr07	13978742	1059	ADT	3.1	161	ETV1	c.365G>A	p.S122N
chr01	45798063	1054	ADT	3.1	63	MUTYH	c.788G>A	p.W263.
chr09	1.34E+08	1028	ADT	3.0	98	NUP214	c.6202T>G	p.F2068V
chr10	30727845	1104	ADT	2.9	102	MAP3K8	c.293A>C	p.D98A
chr03	10134970	1080	ADT	2.9	103	FANCD2	c.3851T>G	p.V1284G
chr03	1.56E+08	1098	ADT	2.9	100	GMPS	c.1982T>G	p.V661G
chr22	29107898	1104	ADT	2.8	71	CHEK2	c.920C>T	p.A307V
chr22	41556647	1080	ADT	2.8	175	EP300	c.3592T>G	p.Y1198D
chr20	54958042	1054	ADT	2.8	142	AURKA	c.565C>T	p.R189W
chr20	57484406	1028	ADT	2.8	142	GNAS	c.2516A>C	p.D839A
chr09	1.24E+08	1050	ADT	2.8	107	CNTRL	c.6956delA	p.E2319fs
chr01	10342447	1040	ADT	2.8	106	KIF1B	c.1290delT	p.H430fs
chr15	66782841	1015	ADT	2.7	111	MAP2K1	c.1070T>G	p.V357G
chr15	67457234	1098	ADT	2.7	147	SMAD3	c.208T>G	p.S70A
chr09	1.34E+08	1040	ADT	2.7	108	NUP214	c.1924A>T	p.S642C
chr04	1941510	1028	ADT	2.7	73	WHSC1	c.1886_1888delAAT	p.K629_630delinsK
chr01	6194779	1028	ADT	2.7	219	CHD5	c.3011T>G	p.V1004G

chr13	48934263	1050	ADT	2.6	115	RB1	c.718A>T	p.K240.
chr05	56170860	1084	ADT	2.6	153	MAP3K1	c.1688T>G	p.V563G
chr17	57752063	1104	ADT	2.5	120	CLTC	c.2423T>G	p.V808G
chr14	95572016	1080	ADT	2.5	78	DICER1	c.3092A>C	p.Q1031P
chr13	1.03E+08	1080	ADT	2.5	159	BIVM	c.1238A>G	p.Q413R
chr12	416113	1040	ADT	2.5	155	KDM5A	c.4073A>T	p.K1358M
chr11	1.08E+08	1080	ADT	2.5	118	ATM	c.3578T>G	p.V1193G
chr03	1.56E+08	1084	ADT	2.5	158	GMPS	c.722T>G	p.V241G
chr02	2.13E+08	1028	ADT	2.5	240	ERBB4	c.1123G>A	p.G375R
chr17	78896525	1054	ADT	2.4	123	RPTOR	c.2522C>A	p.A841D
chr16	16269767	1028	ADT	2.4	163	ABCC6	c.2325G>T	p.R775S
chr08	38275890	1080	ADT	2.4	123	FGFR1	c.1379T>G	p.V460G
chr06	1.18E+08	1104	ADT	2.4	163	ROS1	c.2495delA	p.K832fs
chr02	2.13E+08	1028	ADT	2.4	202	ERBB4	c.1488T>A	p.C496.
chr01	6185160	1084	ADT	2.4	122	CHD5	c.4394G>A	p.R1465K
chr01	1.71E+08	1017	ADT	2.4	121	PRRX1	c.419T>G	p.V140G
chr03	52436896	1080	ADT	2.3	84	BAP1	c.1951T>G	p.F651V
chr01	16247366	1104	ADT	2.3	128	SPEN	c.1637T>G	p.V546G
chr19	15350204	1054	ADT	2.2	87	BRD4	c.3575delA	p.K1192fs
chr15	67457591	1080	ADT	2.2	174	SMAD3	c.401T>G	p.V134G
chr12	46211636	1084	ADT	2.2	89	ARID2	c.602C>A	p.T201N
chr09	1.34E+08	1084	ADT	2.2	88	NUP214	c.1705G>T	p.A569S
chr07	1.29E+08	1080	ADT	2.2	174	SMO	c.1654T>G	p.L552V
chr03	10140501	1080	ADT	2.2	131	FANCD2	c.4283T>G	p.V1428G
chr01	1.51E+08	1080	ADT	2.2	133	SETDB1	c.1268G>T	p.G423V
chr01	2.07E+08	1098	ADT	2.2	227	IKBKE	c.1429T>G	p.F477V
chr12	4398008	1028	ADT	2.1	141	CCND2	c.572A>C	p.D191A
chr10	88991779	1098	ADT	2.1	138	NUTM2A	c.1213T>G	p.F405V
chr10	1.03E+08	1054	ADT	2.1	137	PAX2	c.307T>G	p.Y103D
chr03	1.42E+08	1080	ADT	2.1	142	ATR	c.6318delA	p.K2106fs
chr22	29115384	1098	ADT	2.0	98	CHEK2	c.811A>G	p.S271G
chr16	2111872	1098	ADT	2.0	147	TSC2	c.1393A>C	p.T465P
chr14	74994051	1104	ADT	2.0	149	LTBP2	c.2387A>C	p.Q796P
chr06	1.18E+08	1054	ADT	2.0	146	ROS1	c.6134C>T	p.T2045M
chr01	45798358	1104	ADT	2.0	143	MUTYH	c.578T>G	p.V193G

Supplementary Table S3 - Gene mutations shared between pre-treatment and post-treatment

Chromosome	Position	Patient ID	Treatment	Mutant Allele Frequency % Pre-Treatment	Depth In Pre-Treatment	Mutant Allele Frequency % Post-Treatment	Depth IN Post-Treatment	Gene name	DNA Change	Amino Acid Change
chr10	81465798	1001	Chemotherapy	15.7	57	22.9	74	NUTM2B	c.383C>T	p.A128V
chr23	70320535	1014	Chemotherapy	7.8	51	3.8	77	FOXO4	c.455A>C	p.N152T
chr16	16267139	1043	Chemotherapy	12.8	109	11.4	87	ABCC6	c.2917T>G	p..973E
chr15	99500291	1043	Chemotherapy	2.3	128	3.6	110	IGF1R	c.3724T>G	p.F1242V
chr17	7578553	1028	ADT	2.7	145	2.4	125	TP53	c.377A>C	p.Y126S
chr06	168352869	1028	ADT	3.0	98	2.1	142	MLLT4	c.4766T>G	p.V1589G
chr17	5268418	1050	ADT	2.3	126	3.4	87	RABEP1	c.1670T>G	p.V557G
chr18	60985313	1059	ADT	2.0	149	2.3	130	BCL2	c.587T>G	p.V196G
chr01	164818578	1080	ADT	4.2	95	4.5	87	PBX1	c.1202A>C	p.H401P
chr02	25470026	1104	ADT	2.2	180	4.7	127	DNMT3A	c.1016T>G	p.V339G
chr17	36874099	1104	ADT	4.8	166	2.8	107	MLLT6	c.1916T>G	p.V639G

Supplementary Table S4 - List of all mutated genes in different treatment status

Pre-chemotherapy	Post-chemotherapy	Pre-ADT	Post-ADT
NUTM2B	KDM6A	FOXO4	SH3GL1
FBXO11	MTOR	PIK3CA	KRT17
ARID1A	USP6	ACSL6	EP300
MTOR	KRT17	NDRG1	PTK2
TCF7L2	ERCC3	STK11	PRDM16
TOP1	CRTC1	MYH11	PDGFRB
AKT1	PTK2	NBN	PTPN11
KTN1	CDKN2A	PHF6	CNTRL
FCRL4	APC	TLX1	NSD1
NUP214	PBX1	DNMT3A	STXBP2
CSF1R	NUTM2A	LPP	DEK
PER1	RET	FLCN	TPM4
FLT1	PRKAR1A	NOTCH1	SF3B2
SPEN	PTCH1	PAX3	TFEB
KDM6A	ROS1	RABEP1	KIF1B
PAX8	NTRK1	AFF3	BAP1
FBXW7	KTN1	NUP214	CREB1
MLH1	SMARCB1	RALGDS	CBL
PCSK7	SETD2	UTY	MED12
PBX1	NSD1	ATR	HSP90AB1
NSD1	EPHB6	ARID5B	CNBP
FLCN	TPR	CRTC3	PAX7
FANCA	ELAC2	ATM	ALDH2
MLLT4	PLK1	GUCY1A2	ETV1
NPM1	PBRM1	IKBKE	MUTYH
ATIC	IKBKE	TNFRSF14	NUP214
TPM3	MED12	TRIM27	MAP3K8
NUMA1	MAP3K1	CCNE1	FANCD2
SMARCA4	AKAP9	PMS1	GMPS
TPM4	SF3B2	AIP	CHEK2
DAXX	NFIB	LRP5	AURKA
CHD6	DNMT3A	KMT2A	GNAS
PML	STXBP2	FNBP1	MAP2K1
ROS1	CBLC	LASP1	SMAD3
ERCC3	NFKB2	TET1	WHSC1
TP53	CHCHD7	FANCA	CHD5
HNRNPA2B1	KIF1B	IGF1R	RB1
CIITA	NUP214	CREB1	MAP3K1
STXBP2	NOTCH1	RAD51B	CLTC

NF1	PAX7	ARID5A	DICER1
FGFR1	PATZ1	PRDM16	BIVM
CBL	ABCC6	SH3GL1	KDM5A
WHSC1	PAX2	CCND2	ATM
MKL1	WHSC1	PBRM1	ERBB4
DDIT3	PRKDC	ARID3B	RPTOR
GLMN	PCSK7	CBLC	ABCC6
FGFR1OP	FNBP1	CNTRL	FGFR1
DNMT3A	PRDM1	NF1	ROS1
CHEK2	EPHA6	RET	PRRX1
FOXO4	ETV5	EIF4A2	SPEN
ABCC6	PER1	GAS7	BRD4
IGF1R	ASPSCR1	NIN	ARID2
	FANCG	MYB	SMO
	NCKIPSD	ERCC1	SETDB1
	EPHA10	PTCH1	IKBKE
	MKL1	ERCC5	CCND2
	NTRK2	BIVM-ERCC5	NUTM2A
	PTK2B	WHSC1	PAX2
	KDR	SUZ12	ATR
	PDE4DIP	MLLT6	TSC2
	NUTM2B	ASPSCR1	LTBP2
	FOXO4	CHEK1	TP53
	IGF1R	TP53	MLLT4
		MLLT4	RABEP1
		BCL2	BCL2
		PBX1	PBX1
			DNMT3A
			MLLT6

Supplementary Table S5 - Primer list and PCR conditions

GENES	POSITION	PRIMERS	SEQUENCES	Tm	PRODUCT (bp)	PATIENT ID	Treatment Type	Treatment Status	VALIDATED
NUTM2B	81465798	Forward Primer	5'-TCA TAC GCC CTT AGC TGT TGG-3'	68	119	1001	Chemotherapy	Pre	Yes
		Reverse Primer (mutant)	5'-CGG TCC CAG CAC TGG GTA TA-3'			1001	Chemotherapy	Post	Yes
		Reverse Primer (wild type)	5'-CGG TCC CAG CAC TGG GTA TG-3'			1043	Chemotherapy	Pre	Yes
ABCC6	16267139	Forward Primer	5'-CCT CTG GAT GAC CCT GAC A-3'	64	84	1043	Chemotherapy	Pre	Yes
		Reverse Primer (mutant)	5'-GTG GGT GAA GCT GGT GGT TC-3'			1043	Chemotherapy	Post	Yes
		Reverse Primer (wild type)	5'-GTG GGT GAA GCT GGT GGT TA-3'						
MLLT4	168352869	Forward Primer	5'-ATG GGA AAC ACG CAG AAG C-3'	Uncertain	83	1028	ADT	Pre	Uncertain
		Reverse Primer (mutant)	5'-CTG AAC GAA GAG CGA GGG G-3'			1028	ADT	Post	Uncertain
		Reverse Primer (wild type)	5'-CTG AAC GAA GAG CGA GGG T-3'						
PBX1	164818578	Forward Primer	5'-CTC AGT GTT CTC CTG CTT CG-3'	62	122	1080	ADT	Pre	Yes
		Reverse Primer (mutant)	5'-ACT TAG TCT TCT CTA TAC CCA GCC-3'			1080	ADT	Post	Yes
		Reverse Primer (wild type)	5'-ACT TAG TCT TCT CTA TAC CCA GCA-3'						
DNMT3A	25470026	Forward Primer	5'-GCA AGG CAT GGG GTG GGT-3'	61	89	1104	ADT	Pre	Uncertain
		Reverse Primer (mutant)	5'-GGC ATC AGC TTC TCA ACA CAC C-3'			1104	ADT	Post	Yes
		Reverse Primer (wild type)	5'-GGC ATC AGC TTC TCA ACA CAC A-3'						
FBXO11	48040930	Forward Primer	5'-AAC CCC AAA ATT AGA CGC A-3'	Uncertain	91	1001	Chemotherapy	Pre	Uncertain
		Reverse Primer (mutant)	5'-AAA AAG ATG ACA GAT TAA ACA TAC T-3'						
		Reverse Primer (wild type)	5'-AAA AAG ATG ACA GAT TAA ACA TAC C-3'						
ACSL6	131325794	Forward Primer	5'-TGC CCA CAT CCC TCC CTA C-3'	Uncertain	99	1080	ADT	Pre	Uncertain
		Reverse Primer (mutant)	5'-CAG TGG CTG TCC TAC CAG GG-3'						
		Reverse Primer (wild type)	5'-CAG TGG CTG TCC TAC CAG GA-3'						
TCF7L2	114711242	Forward Primer	5'-CCC TCG GGG CAC TTT CTA A-3'	61	106	1002	Chemotherapy	Pre	Yes
		Reverse Primer (mutant)	5'-TCC ATC TTG CCT CTT GGC CA-3'						
		Reverse Primer (wild type)	5'-TCC ATC TTG CCT CTT GGC CG-3'						
CSF1R	149437068	Forward Primer	5'-GGT GGG AAG AGG CGT CAG-3'	66	139	1002	Chemotherapy	Pre	Yes
		Reverse Primer (mutant)	5'-CAA ATG ACT CCT TCT CTG AGC AT-3'						
		Reverse Primer (wild type)	5'-CAA ATG ACT CCT TCT CTG AGC AA-3'						
FLT1	28979918	Forward Primer	5'-ATA AAC CTA GAA TTG GGA GCT G-3'	59	99	1002	Chemotherapy	Pre	Yes
		Reverse Primer (mutant)	5'-TGG CAA TAA TAG AAG GAA AGA ATA G-3'						
		Reverse Primer (wild type)	5'-TGG CAA TAA TAG AAG GAA AGA ATA A-3'						
MLLT4	168291542	Forward Primer	5'-GCC ATC CTG ACC AAC CTG A-3'	Uncertain	81	1001	Chemotherapy	Pre	Uncertain

		Reverse Primer (mutant)<br>Reverse Primer (wild type)	5'-TCT CTT CAA CTG AAA GAC TAA AAT CA-3'<br>5'-TCT CTT CAA CTG AAA GAC TAA AAT CC-3'						
LRP5	68177382	Forward Primer<br>Reverse Primer (mutant)<br>Reverse Primer (wild type)	5'-TGC CCA TCC AGT CAA CGG-3'<br>5'-CTC CTC ACC TGC TGC CAG C-3'<br>5'-CTC CTC ACC TGC TGC CAG A-3'	61	115	1028<br>1098	ADT<br>ADT	Pre<br>Pre	Yes<br>Yes
AKT1	105239429	Forward Primer<br>Reverse Primer (mutant)<br>Reverse Primer (wild type)	5'-TCC CGG ACA CCC CTT GAT G-3'<br>5'-CCG TAG TCA TTG TCC TCC AGC AT-3'<br>5'-CCG TAG TCA TTG TCC TCC AGC AC-3'	Uncertain	72	1005<br>1017	Chemotherapy<br>Chemotherapy	Pre<br>Post	Uncertain<br>Uncertain
NUP214	134015937	Forward Primer<br>Reverse Primer (mutant)<br>Reverse Primer (wild type)	5'-ATG TTG AGG GCA GTC TTT G-3'<br>5'-GGA GGA AGA GTC TTT TCA TCC-3'<br>5'-GGA GGA AGA GTC TTT TCA TCA-3'	56	103	1002<br>1028	Chemotherapy<br>ADT	Pre<br>Pre	Yes<br>Yes
EP300	41531818	Forward Primer<br>Reverse Primer (mutant)<br>Reverse Primer (wild type)	5'-TTG TAT GGT GGC TGT TGT ATT TAT T-3'<br>5'-CCA TTT ACT CCC ATA GGA CTA GCC-3'<br>5'-CCA TTT ACT CCC ATA GGA CTA GCA-3'	63	80	1028	ADT	Post	Yes
PTK2	141900643	Forward Primer<br>Reverse Primer (mutant)<br>Reverse Primer (wild type)	5'-TAT GAA AAG TCC CCG ATA AGT T-3'<br>5'-CAT GGA GAT GCT ACT GAT GTC AA-3'<br>5'-CAT GGA GAT GCT ACT GAT GTC AG-3'	62	139	1080	ADT	Post	Yes
PTK2	141716219	Forward Primer<br>Reverse Primer (mutant)<br>N/A (wild type)	5'-ATG CAC AAT GTA CCG CTC TAC C-3'<br>5'-GCA CAT GGT ACA AAC CAA TCA GG-3'	63	97	1002	Chemotherapy	Post	Yes
PRKAR1A	66523982	Forward Primer<br>Reverse Primer (mutant)<br>Reverse Primer (wild type)	5'-ATT CCA TAG CAT TAT GTG GTG AT-3'<br>5'-TTC CGC TTT CTC AGT GTG CTT A-3'<br>5'-TTC CGC TTT CTC AGT GTG CTT C-3'	52	91	1005	Chemotherapy	Post	Yes
ROS1	117642422	Forward Primer<br>Reverse Primer (mutant)<br>Reverse Primer (wild type)	5'-TAC TGT TGC CCA CCC TTT GC-3'<br>5'-CTA ATG CCT GCT ATG CAA TAC G-3'<br>5'-CTA ATG CCT GCT ATG CAA TAC A-3'	67	77	1017	Chemotherapy	Post	Yes
SMARCB1	24143268	Forward Primer<br>Reverse Primer (mutant)<br>Reverse Primer (wild type)	5'-CTC CCA CCA CTT AGA TGC CGT-3'<br>5'-TGC AGC GAT GCA TCC ACA CA-3'<br>5'-TGC AGC GAT GCA TCC ACA CC-3'	Uncertain	106	1017	Chemotherapy	Post	Uncertain
ABCC6	16269767	Forward Primer<br>Reverse Primer (mutant)<br>Reverse Primer (wild type)	5'-GCC TAA CTG CCC GAG ATG C-3'<br>5'-CCG AGC TTA GAC GCG AGA GT-3'<br>5'-CCG AGC TTA GAC GCG AGA GG-3'	67	106	1028	ADT	Post	Yes
PLK1	23693386	Forward Primer<br>Reverse Primer (mutant)<br>Reverse Primer	5'-GGG TTG TGG CTG GGA GAC TG-3'<br>5'-TGG TTT GCC CAC TAA CAA GGT ATC-3'<br>5'-TGG TTT GCC CAC TAA CAA	67	74	1001<br>1043	Chemotherapy<br>Chemotherapy	Post<br>Post	Yes<br>Yes

		(wild type)	GGT ATA-3'						
ERCC3	128017023	Forward Primer	5'-GGA CCC AGG AGA AGG CAG AG-3'	68	96	1001	Chemotherapy	Post	Yes
		Reverse Primer (mutant)	5'-GCC AGC GAG TTT CGT GAT CC-3'			1017	Chemotherapy	Post	Yes
		Reverse Primer (wild type)	5'-GCC AGC GAG TTT CGT GAT CA-3'						
EPHB6	142561389	Forward Primer	5'-CCA AGG GAT TCA GGT TCA GA-3'	59	121	1003	Chemotherapy	Post	Yes
		Reverse Primer (mutant)	5'-CCC TCT TAT TTC TGG GCA GG-3'			1017	Chemotherapy	Post	Yes
		Reverse Primer (wild type)	5'-CCC TCT TAT TTC TGG GCA GA-3'						
MED12	70354208	Forward Primer	5'-ACT CCG TGG TCT GCT GGG TGC T-3'	61	70	1010	Chemotherapy	Post	Yes
		Reverse Primer (mutant)	5'-TTG TTG TGG CCC TGG CAG GG-3'			1043	Chemotherapy	Post	Yes
		Reverse Primer (wild type)	5'-TTG TTG TGG CCC TGG CAG GT-3'			1098	ADT	Post	Yes
CRTC1	18856633	Forward Primer	5'-CAC GCT CCC GGT ACA CCC TG-3'	58	101	1001	Chemotherapy	Post	Yes
		Reverse Primer (mutant)	5'-CCA TCT CCT CCT CCC CCA GC-3'			1017	Chemotherapy	Post	Yes
		Reverse Primer (wild type)	5'-CCA TCT CCT CCT CCC CCA GA-3'						
